# Impact of Iron Deficiency on the *Arabidopsis thaliana* Phloem Sap Proteome, a Key Role for bHLH121

**DOI:** 10.1111/ppl.70336

**Published:** 2025-06-19

**Authors:** Berger Nathalie, Kalra Muskan, Gao Fei, Rofidal Valérie, Demolombe Vincent, Santoni Véronique, Dubos Christian

**Affiliations:** ^1^ IPSiM, CNRS, INRAE, Institut Agro, University Montpellier Montpellier France; ^2^ College of Agronomy, Hunan Agricultural University Changsha China; ^3^ Yuelushan Laboratory Changsha China

**Keywords:** Arabidopsis, bHLH121, iron, phloem, proteomics

## Abstract

Iron (Fe) is an essential micronutrient for plant growth and development whose homeostasis must be tightly regulated to avoid deficiency or excess that could be detrimental to the cells. In *Arabidopsis thaliana* , this mechanism is regulated by a series of transcription factors that act in an intricate regulatory network among which URI/bHLH121 (UPSTREAM REGULATOR OF IRT1) plays a predominant role. Tremendous efforts were deployed to decipher the molecular mechanisms that regulate iron homeostasis in plants. Nonetheless, the nature of the long‐distance signal that conveys, via the phloem sap, information on the iron status of aerial tissues to the roots in order to coordinate iron uptake with the plant needs for iron is still to be determined. With the aim to identify potential actors involved in this process, we set up a proteomic analysis of the phloem sap of wild type Arabidopsis plants and *bhlh121* loss‐of‐function mutants grown in iron‐replete and iron‐deficient conditions. We found that modifications in iron availability or the loss of URI activity have a profound impact on the phloem sap protein composition. We also found that some proteins whose translocation through the phloem sap is inhibited in response to iron deficiency are also affected in *bhlh121* mutants. Interestingly, we discovered that some of the genes encoding such proteins are direct targets of URI, which suggests that the encoded proteins might act as potential signaling factors to regulate root iron uptake and/or root growth.

## 1 Introduction

Iron (Fe) is an essential micronutrient for plant growth and development (Briat et al. 2015). Due to its redox‐active nature, iron is a cofactor for several metalloproteins involved in essential physiological mechanisms such as respiration or the biosynthesis of chlorophylls and amino acids (Balk and Pilon 2011; Chen and Browne 2018; Touraine et al. 2019). Nonetheless, iron excess can be deleterious to the cell because of its capacity to react with oxygen, producing reactive oxygen species (ROS; Connolly and Guerinot 2002). Consequently, cell iron homeostasis must be tightly regulated.

Most of the iron present in the soil is in the form of Fe^3+^ oxides/hydroxides which are hardly available for plants. Consequently, plants have developed sophisticated strategies to cope with this low iron availability. Non‐grass species use the concomitant activity of H^+^‐ATPASES and FERRIC REDUCTASES to solubilize and reduce Fe^3+^ to Fe^2+^ that is then taken up from the rhizosphere into the root cells by high iron‐affinity transporters from the ZRT/IRT‐LIKE PROTEIN (ZIP) family. In *Arabidopsis thaliana* , these three steps are under the control of AHA2 (H^+^‐ATPASE 2), FRO2 (FERRIC REDUCTION OXIDASE 2), and IRT1 (IRON REGULATED TRANSPORTER 1), respectively (Robinson et al. 1999; Vert et al. 2002; Santi and Schmidt 2009). In addition, it has emerged that the secretion of iron‐mobilizing coumarins by the root play an important role in this process (Fourcroy et al. 2014; Schmidt et al. 2014; Sisó‐Terraza et al. 2016; Rajniak et al. 2018; Siwinska et al. 2018; Tsai et al. 2018; Vanholme et al. 2019; Robe, Izquierdo, et al. 2021; Paffrath et al. 2024).

Following its uptake, iron translocates from roots to photosynthetic organs via the xylem in the form of Fe^3+^‐citrate chelates. This process requires the activity of the FRD3 (FERRIC REDUCTASE DEFECTIVE 3) citrate transporter (Rogers and Guerinot 2002). Conversely, iron can be remobilized and translocated to sink tissues (i.e., young leaves, pollen, seeds, or roots) in the form of Fe^2+^‐nicotianamine complexes through the phloem, thanks to the activity of OPT3 (OLIGOPEPTIDE TRANSPORTER 3) and transporters of the YSL (YELLOW STRIPE LIKE) family (Stacey et al. 2008; Zhai et al. 2014). Interestingly, the *opt3* mutation (loss‐of‐function) causes a constitutive activation of the iron acquisition machinery even in iron‐sufficient growth conditions. Such observation emphasizes the role played by the phloem in transmitting leaves' iron status to the roots to modulate the activity of the iron uptake machinery, and the role of OPT3 in this long‐distance signaling process (Chia et al. 2023).

The modulation of iron uptake, transport, and partitioning relies on the combined activity of several transcription factors that act in an intricate regulatory network, among which bHLHs play a dominant role (Gao and Dubos 2021; Li et al. 2023; Gao and Dubos 2024; Liang et al. 2022; Trofimov et al. 2025). In this network, FIT/bHLH29 (FER‐LIKE IRON DEFICIENCY INDUCED TRANSCRIPTION FACTOR) heterodimerized with the four clade Ib bHLH to positively regulate the expression of *IRT1* and *FRO2*, and thus the uptake of iron (Colangelo and Guerinot 2004; Jakoby et al. 2004; Yuan et al. 2005). *FIT* and clade Ib bHLH expression is itself regulated by another set of positive regulators of the bHLH family, among which the master regulator URI/bHLH121 (UPSTREAM REGULATOR OF IRT1) acts synergistically with clade IVc bHLH (Kim et al. 2019; Gao, Robe, Bettembourg, et al. 2020; Lei et al. 2020). The stability of these bHLHs (e.g., FIT, URI) is regulated by a hemerythrin domain containing E3‐ubiquitin ligases, whose activity is inhibited by their interaction with small proteins composed of about 60 amino acids named IMAs/FEPs (IRON MAN/FE UPTAKE INDUCING PEPTIDES; Selote et al. 2015; Grillet et al. 2018; Li et al. 2021). It is noteworthy that the expression of these E3‐ubiquitin ligases and *IMAs* is also under the control of URI.

Even if tremendous efforts have been deployed during the last two decades to decipher the molecular mechanisms that regulate iron homeostasis in plants, the nature of the long‐distance signal that conveys information of the iron status of aerial tissues to the roots in order to coordinate root growth and iron uptake with the plant's needs for iron via the phloem sap is still unclear (Grusak and Pezeshgi 1996). It has been demonstrated that small molecules, RNAs, peptides, or proteins can be transported via the xylem and phloem saps, allowing long‐distance signaling of several physiological or environmental cues (Takahashi and Shinozaki 2019). For instance, the FT protein (FLOWERING LOCUS T), identified as a component of the florigenic signal in angiosperms, is translocated through the phloem to the shoot apex where it induces flowering (Corbesier et al. 2007; Lin et al. 2007). Other studies have highlighted that proteins involved in stress and defense responses are also present within the phloem translocation stream (Walz et al. 2004; Castaneda et al. 2021). HY5 (ELONGATED HYPOCOTYL 5), a bZIP transcription factor implicated in photomorphogenesis, coordinates plant carbon and nitrogen acquisition by acting in shoot‐to‐root signaling (Mankotia et al. 2024). HY5 is also implicated in iron homeostasis regulation (Mankotia et al. 2025). Similarly, IMAs were associated with long‐distance signaling of leaves' iron status to roots and proposed to act as phloem‐mobile signals (Grillet et al. 2018; Hirayama et al. 2018). Nonetheless, the relationship between iron long‐distance signaling and IMAs is still unclear since their phloem translocation has not been demonstrated.

To get further insights into the potential involvement of proteins in long‐distance signaling of leaves’ iron status to the root system, a label‐free proteomic experiment was done to determine the phloem sap protein composition in wild type (WT) and knockout Arabidopsis mutants of the master regulator gene *URI* (i.e., *bhlh121*). This study first revealed that modifications in iron availability or the loss of URI activity have a profound impact on the phloem sap protein composition. Furthermore, we found that some proteins whose translocation through the phloem sap is inhibited in response to iron deficiency are also affected in *bhlh121* mutants. Interestingly, we found that genes encoding such proteins are direct targets of URI, which suggests that the encoded proteins might act as potential signaling factors to regulate root iron uptake and/or root growth.

## 2 Material and Methods

### 2.1 Plant Material and Growth Conditions

For proteomic analysis and RNA quantification by RT‐qPCR, plants were grown under short day conditions (8 h/16 h and 23°C/20°C, day/night) at a light intensity of 160 μmol m^−2^ s^−1^ and 65% humidity. *Arabidopsis thaliana* WT (Col‐0) and *bhlh121‐2* (URI loss‐of‐function mutant; Gao, Robe, Bettembourg, et al. 2020) seeds were surface sterilized and sown in 0.2 mL tubes containing 0.8% agar prepared in pH 5.5 Hoagland solution with 50 μM Fe‐EDTA (control Fe condition). After 7 days of growth, the bottoms of the tubes were cut off, allowing root growth outside of the tubes, prior to transfer to 2.5 L opaque recipients containing the same Hoagland medium (Robe et al. 2020; Robe, Conejero, et al. 2021). Following 6 weeks of growth, half of the plants of each genotype were then transferred in control Hoagland solution (+Fe WT and + Fe *bhlh121*) or Hoagland solution deprived of iron (−Fe WT and −Fe *bhlh121*) for 6 days.

For the ChIP‐qPCR experiment, plants were grown under long day conditions (16 h/8 h and 23°C/20°C, day/night) at a light intensity of 160 μmol m^−2^ s^−1^ and 65% humidity. Arabidopsis transgenic lines carrying the *ProbHLH121:GFP* and *ProbHLH121:gbHLH121‐GFP* constructs (Gao, Robe, Bettembourg, et al. 2020) were surface sterilized and sown on square petri dishes containing 0.8% agar prepared in half Murashige and Skoog (MS/2) media with 50 μM Fe‐EDTA (control condition). After 7 days of growth, half of the plants were transferred to MS/2 media with 50 μM Fe‐EDTA and the other half to MS/2 media without iron. Plants were harvested 4 days after the transfer.

### 2.2 Sample Preparation for Label‐Free Proteomic Analysis

2o leaves of three plants (three independent replicates) per genotype and per condition were harvested for a phloem exudation experiment according to Tetyuk et al. (2013). The 20 leaves of a given plant were treated together (one replicate). Petioles were placed in 20 mM K_2_‐EDTA solution for 1 h and rinsed with Milli‐Q water. Phloem sap was collected by plunging the petiole of the leaves in tubes containing Milli‐Q water supplemented with a commercial antiprotease mix (cOmplete Tablets, Mini EDTA‐free, EASYpack, Roche) according to the manufacturer's instructions. After collection, phloem exudates were frozen at −20°C, lyophilized, and solubilized in 50 μL of 1 X Laemmli solution (65 mM Tris–HCl, pH 7.5, 5% glycerol, 2% SDS; Laemmli 1970). 10 μL of each sample were loaded on a 10% pre‐cast gel (Bio‐Rad) and subjected to a 20 min migration at 100 V. Each line of the gel was cut into three parts and each part into small pieces (2 mm^2^). Then, the following steps of the sample preparation were done according to Berger et al. (2020). Each sample was solubilized in 7 μL of 2% formic acid and 2.5 μL was injected for a 150 min run of LC‐MS/MS analysis (Qexactive Plus, ThermoFisher Scientific).

### 2.3 Mass Spectrometry Data Processing for Protein Identification and Quantification

Raw data were analyzed using the Maxquant software v2.0.3.0 with default parameters except that the minimum score was set to 20 and 40 for unmodified and modified peptides, respectively (Cox and Mann 2008). Variable modifications included in protein identification and quantification were oxidation (M), acetyl (Protein *N*‐term), and phospho (STY). Proteins were identified and quantified only if two peptides (at least one unique peptide) were identified in the Araport_genes_20210122 database with Andromeda (Cox et al. 2011). Following the quantification step and LFQ normalization, proteins were filtered and considered as identified and quantifiable only if they were present in the three replicates. For statistics, LFQ analyst was used with default parameters (Shah et al. 2020). For this analysis, all LFQ intensities were converted to a log2 scale and LFQ analyst used the Bioconductor package limma to carry out the differential quantification test using the information provided in the experimental design table. Adjusted *p* value cutoff and Log2 fold change cutoff was 0.05 and 1, respectively. The false discovery rate (FDR) correction option used was the Benjamini Hochberg (BH) method. For the label‐free approach on rosettes, the same pipeline was used except the Log2 fold change cutoff that was set to 0.5. For qualitative studies (absent/present), analyses were done with LFQ analyst with “min” imputation type.

### 2.4 ChIP Experiment

Experiments were performed as previously described (Gao, Robe, Bettembourg, et al. 2020; Gao and Dubos 2023). The primers used are described in Supplementary Table S1.

### 2.5 Gene Expression Analysis

Total RNAs were extracted using the nucleospin RNA extraction kit (Macherey‐Nagel). For each sample, 1 μg of total RNA treated with DNase was reverse transcribed into cDNA using the RevertAid kit (Thermo scientific). qRT‐PCR analyses were performed using a LightCycler 480 (Roche) and TB Green Premix Ex Taq (2X; Takara). *PP2AA3* (*PROTEIN PHOSPHATASE 2A SUBUNIT A3*) was used as a reference gene (Czechowski et al. 2005). Expression levels were calculated using the comparative threshold cycle method. The primers used are described inTable S1.

## 3 Results

### 3.1 Phloem Sap Protein Composition of Wild‐Type Plants Grown in Control Conditions

Proteomic analysis of the phloem sap of WT plants grown in iron sufficiency (+Fe) led to the identification of a total of 1414 proteins (+Fe WT; Data S1). We first compared this protein list with two available *Arabidopsis thaliana* phloem sap proteomes obtained using the same phloem sap extraction protocol from plants grown in iron‐replete conditions (Batailler et al. 2012; Carella et al. 2016). A total of 377 and 611 proteins were identified in the Batailler et al. (2012) and Carella et al. (2016) studies, respectively, which represent about 27% and 43% of the total number of proteins identified in our experiment.

**FIGURE 1:**
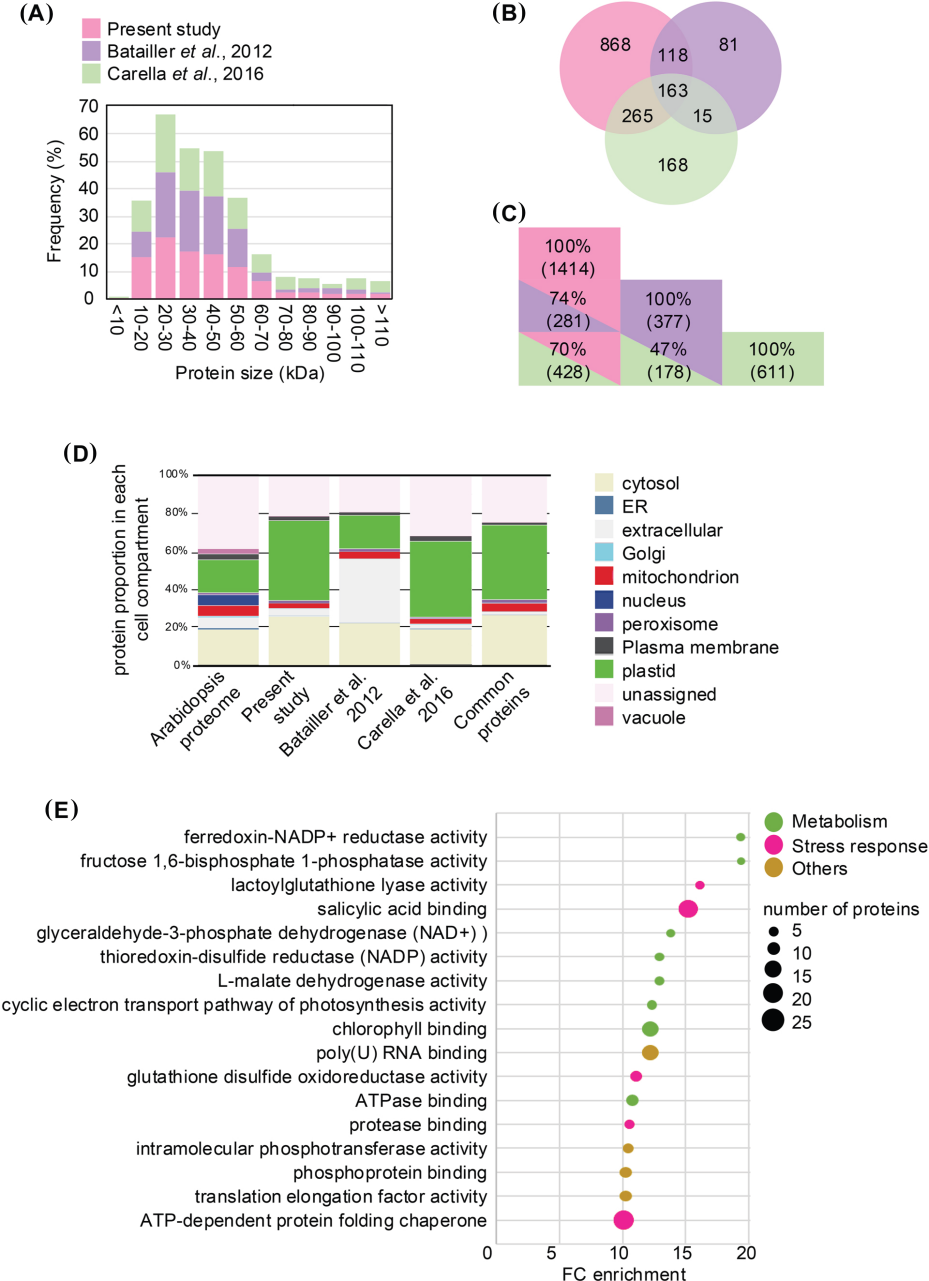
Comparison of phloem sap proteomes of wild‐type plants grown in iron sufficiency. (A) Comparison of the size repartition of the proteins between the present study and the Batailler et al. (2012) and Carella et al. (2016) studies. (B) and (C) Similarities between the protein lists of the three studies. Repartition of the cellular location of proteins found in the whole *Arabidopsis thaliana* genome, the present study, the Batailler et al. (2012) study, the Carella et al. (2016) study, and the 163 proteins common to the three studies. Analysis was done at the following URL: https://suba.Live/toolbox.html (D). GO term enrichment analysis of the proteins found in our study was conducted using the PANTHER gene ontology platform at the following URL: http://Go.pantherdb.org.

We then analyze the protein size distribution in the three studies and found no difference (Figure 1A). Importantly, a predominance of proteins in the range of 20 to 60 kDa was observed, which is in adequacy with the exclusion size of plasmodesmata. Comparison of the protein lists of the two previous studies highlighted that 74% (281) and 70% (428) of proteins identified in the Batailler et al. (2012) and Carella et al. (2016) studies, respectively, were also found in the present phloem sap proteome analysis (Figure 1B,C). Surprisingly, only 47% of the proteins are shared between the Batailler et al. (2012) and Carella et al. (2016) studies and only 163 are in common between the three studies.

Compared to the predicted protein proportion in each cell compartment for the whole set of proteins encoded in the Arabidopsis genome, we found that, as in Batailler et al. (2012), the most enriched compartment in the present study was the chloroplast (Figure 1D). In contrast, it is the extracellular compartment that is overrepresented in the Carella et al. (2016) proteome, which might explain the low proportion of common proteins with the Batailler et al. (2012) study. Nonetheless, in the three phloem sap proteome studies, endoplasmic reticulum, nucleus, and vacuolar proteins are underrepresented. Gene ontology (GO) term enrichment analysis of the 1414 proteins identified in the (+Fe WT) samples indicates that proteins with a predicted function associated with general metabolism (e.g., photosynthesis, TCA cycle), stress response, and RNA and protein processing (Figure 1E) are overrepresented. Such enrichment was similar to that of proteins identified in the Batailler et al. (2012) and Carella et al. (2016) proteomes (Supplementary Data S2).

### 3.2 Phloem Sap Proteome Comparison of WT Plants Grown in Contrasted Iron Conditions

**FIGURE 2:**
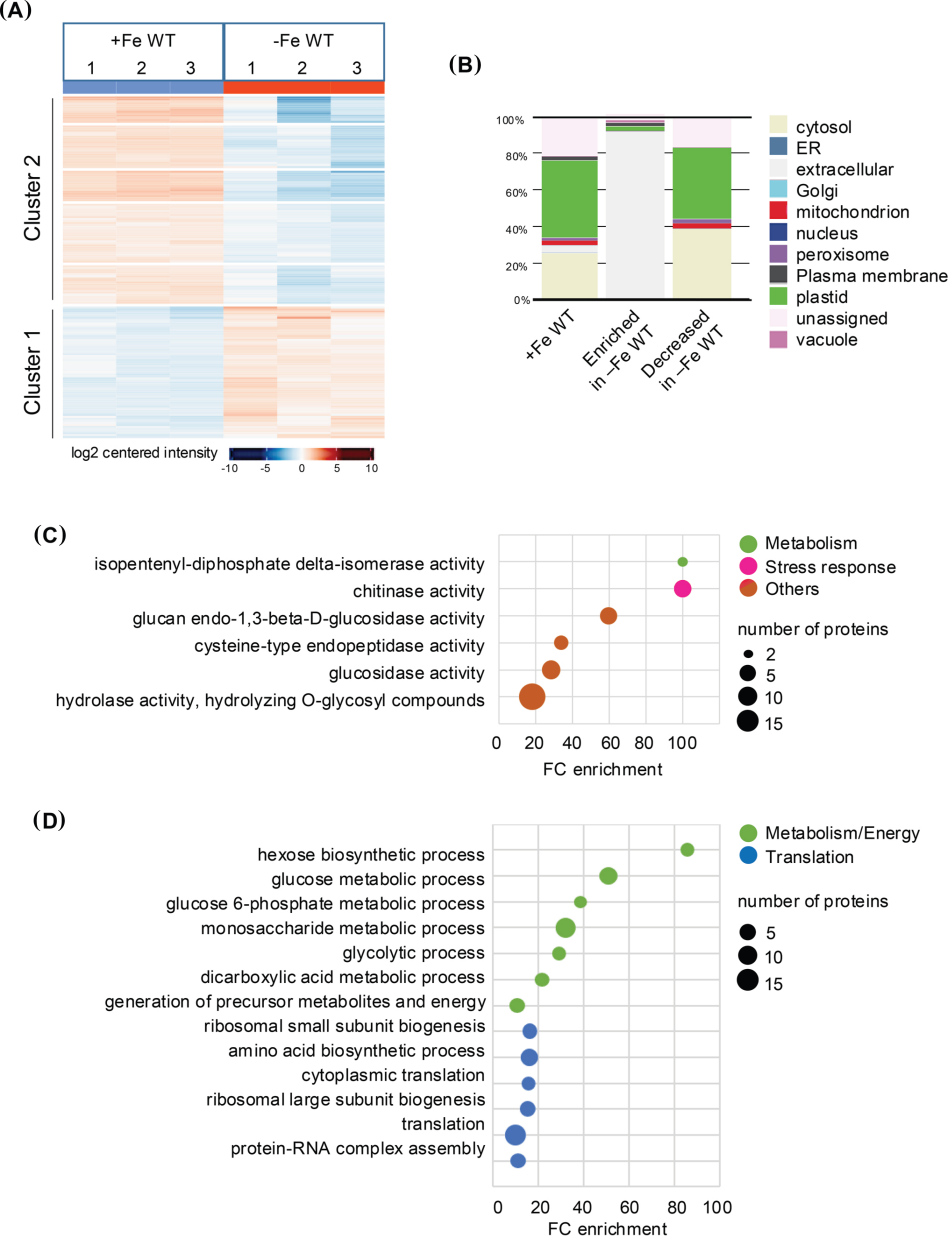
Comparison of phloem sap proteomes of wild‐type plants grown in iron‐sufficient and iron‐deficient conditions. (A) Heat map representation of the three replicates for proteins displaying significant quantitative variation between wild type plants grown under iron‐replete (+Fe WT) and wild type plants grown under iron‐deficient conditions (−Fe WT). The heat map was generated at the following URL: https://analyst‐suite.monash‐proteomics.cloud.edu.au/apps/lfq‐analyst/. (B) Repartition of the cellular location of proteins found in (+Fe WT) and of proteins enriched or under accumulated in response to iron deficiency (−Fe WT). Analyses were done at the following URL: https://suba.Live/toolbox.html. (C) and (D) GO term enrichment analysis for the proteins whose accumulation was increased or decreased in (−Fe WT) when compared to (+Fe WT). Analyses were done using the PANTHER gene ontology platform at the following URL: http://Go.pantherdb.org.

The phloem sap proteome of WT plants grown under iron‐sufficient conditions (+Fe WT) was compared to the one of WT plants grown under iron‐deficient conditions (−Fe WT). A total of 692 proteins identified in all three replicates in each condition were considered for quantitative protein analysis. Among them, the accumulation of 104 and 58 proteins was decreased and increased in response to iron deficiency, respectively (Figure 2A). Qualitative variation, which correspond to proteins identified in the three replicates of one condition and not in the other three replicates of the other condition, were also observed. This is altogether 237 and five proteins that were not identified in (−Fe WT) and + Fe WT, respectively (Data S3).

The protein set whose accumulation is higher in iron deficiency (341 proteins) is highly enriched in extracellular proteins, which represents 84% enrichment compared to the whole Arabidopsis proteome, and 44% compared to the total phloem sap one (Figure 2B). For the protein set with a lower accumulation in iron‐deficient conditions (63 proteins), cytoplasmic proteins were more enriched in the phloem sap proteome compared to the phloem sap reference (+Fe WT).

GO term analysis enrichment on iron deficiency‐enriched proteins highlighted functions related to callose degradation (i.e., β‐glucosidase activity) that are associated to the extracellular location found in the subcellular localization (Figure 2C). For those whose accumulation is decreased in response to iron deficiency, GO term enrichment was observed for functions related to general metabolism and energy as well as the translation process (Figure 2D). It is noteworthy that translation‐related GO terms gather essentially ribosomal proteins (42 proteins in total; Data S4). Interestingly, it was previously reported that the expression of 81 ribosomal proteins was decreased in the roots of plants subjected to iron deficiency (Wang et al. 2013). We found that 16 ribosomal proteins are shared between the two analyses (Data S4) suggesting that the accumulation of these proteins can occur in an iron‐ and tissue‐specific manner.

### 3.3 Proteome Analysis of Rosette Leaves of Wild‐Type Plants Grown in Contrasted Iron Conditions

To better assess the specificity of the phloem sap proteome we have generated, we conducted a global label‐free proteomic experiment on rosette leaves of WT plants cultivated in the same condition than the plants used for phloem sap extraction, in iron‐sufficient and iron‐deficient conditions. It leads to the identification of 3583 proteins among which only nine were associated with iron homeostasis (Data S5). A total of 21 and six proteins displayed a decreased and increased accumulation in iron deficiency (−Fe) conditions, respectively. In addition, 33 and 15 proteins were absent and present in −Fe condition, respectively.

The protein set whose accumulation is decreased in response to Fe deficiency is predominantly composed of plastidial and extracellular proteins (60% and 24%, respectively). Conversely, the protein set whose accumulation is increased in response to Fe deficiency is composed of plastidial (13%), extracellular and plasma membrane proteins (13% each), and cytosolic proteins (26%). As expected, FERRITIN protein (i.e., FER1 and FER3) accumulation was higher in control conditions than in Fe deficiency, whereas the accumulation of NAS4 and OPT3 displayed an opposite trend. Importantly, only four proteins whose accumulations were modified in response to iron availability in the phloem sap proteome were retrieved as differentially accumulated in rosette leaves, confirming the specificity of the phloem sap proteome.

### 3.4 Comparison of the Wild‐Type and bhlh121 Phloem Sap Proteome in Response to Iron Deficiency

The bHLH121 transcription factor is a master regulator of iron homeostasis and consequently *bhlh121* loss‐of‐function mutants display a hypersensitive phenotype to iron deficiency when compared to WT plants. Because of its central role in the transcriptional regulatory network that controls iron homeostasis in Arabidopsis, one might hypothesize that bHLH121 participates in the regulation of the shoot‐to‐root iron status signal. In support of this hypothesis, it has been shown that the expression of the phloem‐associated *IMA* peptides (small proteins) is under the direct regulation of bHLH121.

**FIGURE 3:**
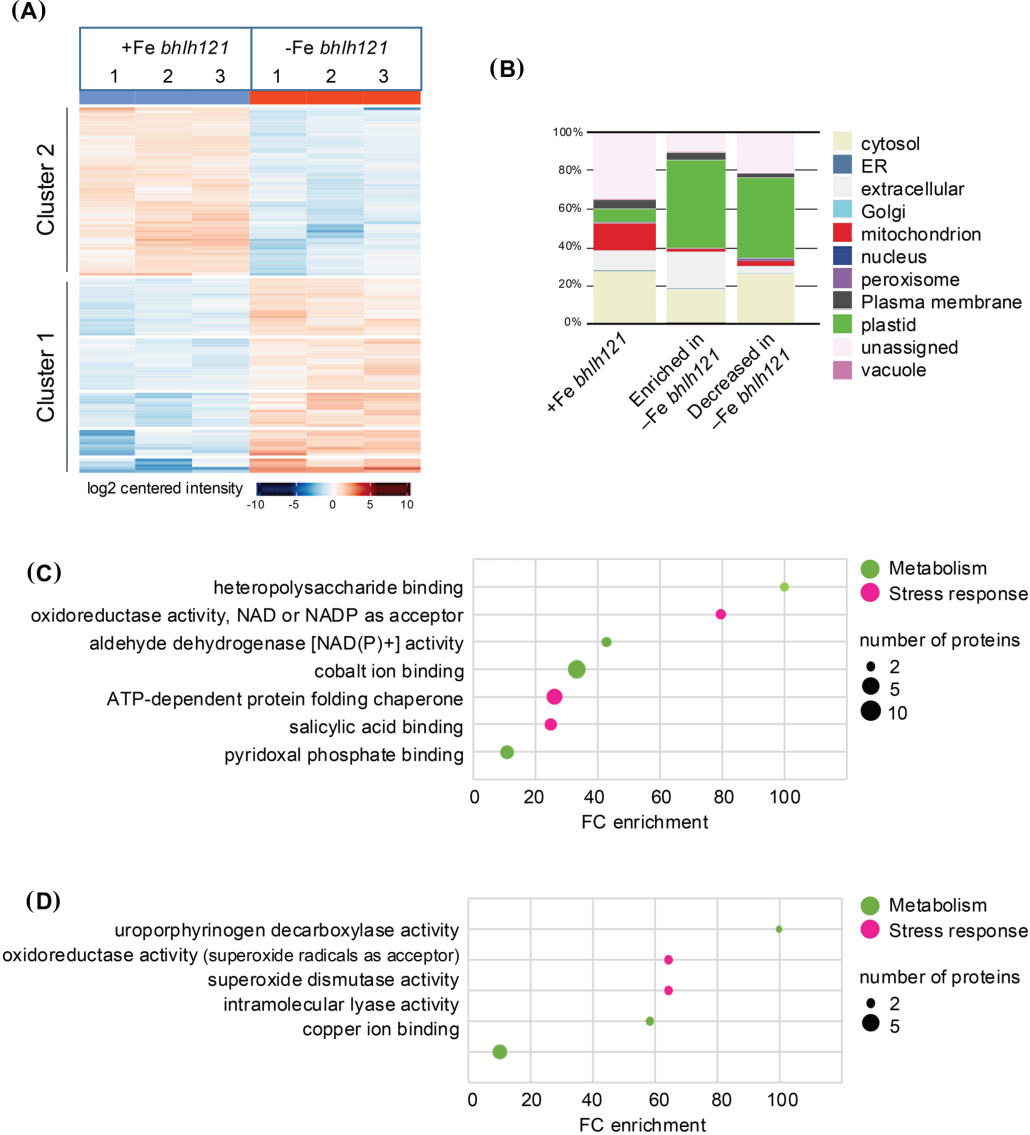
Comparison of phloem sap proteomes of *bhlh121* mutant plants grown in iron‐sufficient and iron‐deficient conditions. (A) Heat map representation of the three replicates for proteins displaying significant quantitative variation between *bhlh121* mutant plants grown under iron‐replete (+Fe *bhlh121*) and *bhlh121* mutant plants grown under iron‐deficient conditions (−Fe *bhlh121*). The heat map was generated at the following URL: https://analyst‐suite.monash‐proteomics.cloud.edu.au/apps/lfq‐analyst/. (B) Repartition of the cellular location of proteins found in +Fe *bhlh121* and of proteins enriched or under accumulated in response to iron deficiency (−Fe *bhlh121*). Analyses were done at the following URL: https://suba.Live/toolbox.html. (C) and (D) GO term enrichment analysis for the proteins whose accumulation was increased or decreased in (−Fe *bhlh121*) when compared to (+Fe *bhlh121*). Analyses were done using the PANTHER gene ontology platform at the following URL: http://Go.pantherdb.org.

Therefore, the phloem sap proteome of *bhlh121* mutants grown in contrasted iron nutrition was generated and analyzed. A total of 94 and 106 proteins displayed a decreased and increased accumulation in iron‐deficient conditions, respectively (Figure 3A). In addition, 30 and 102 proteins were absent and present in iron‐deficient conditions, respectively (Data S3). The 208 proteins whose accumulation is increased in response to iron deficiency were mostly composed of plastidial (45%), extracellular (18%), and cytosolic (18%) proteins (Figure 3B). For those whose accumulation was decreased (124 proteins), the predominant protein classes were plastidial (40%) and cytosolic (30%). GO term analysis revealed that most of these proteins are associated with an enrichment in metabolism, most probably associated with stress responses (Figure 3C,D).

**FIGURE 4:**
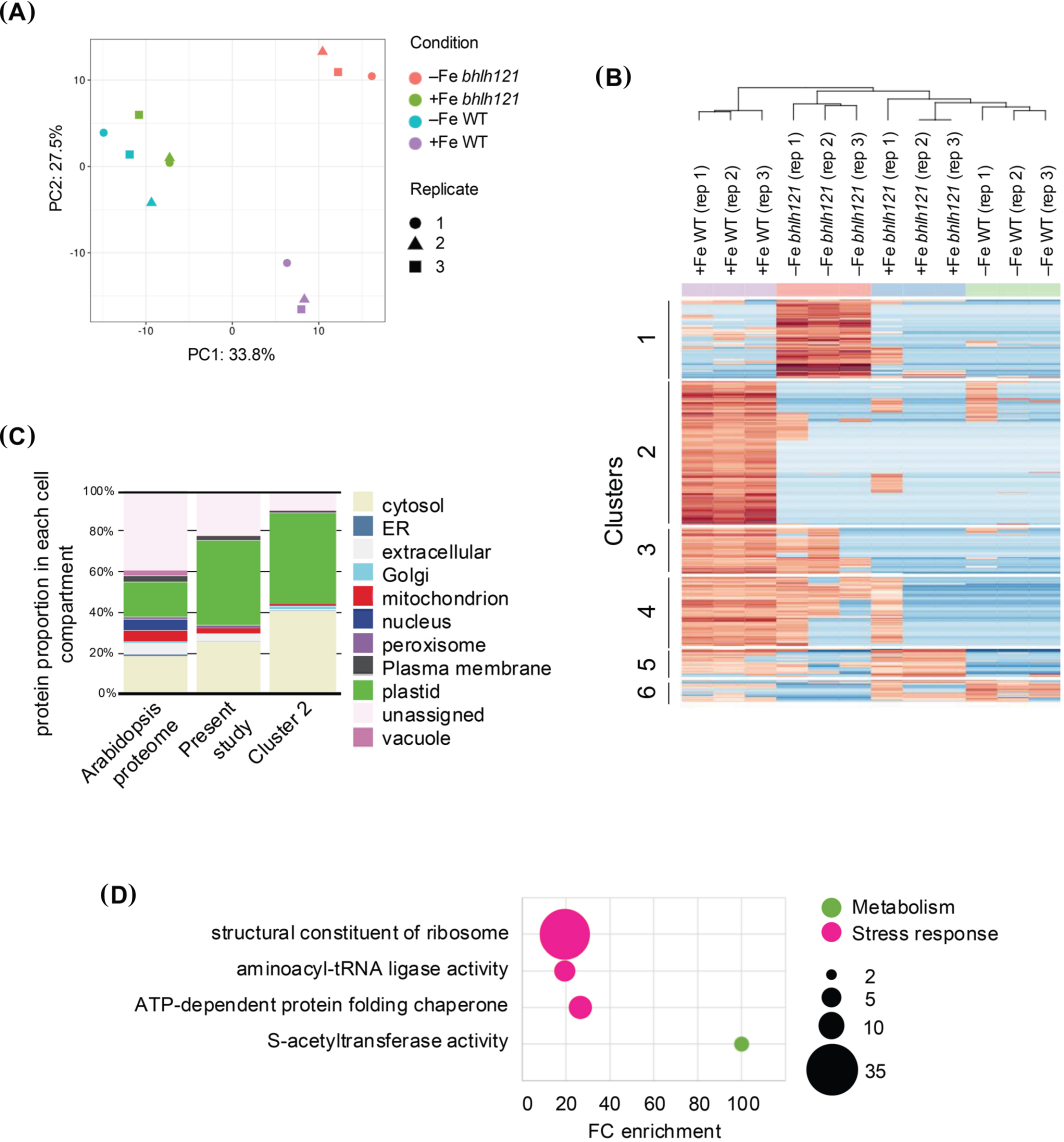
Comparison of phloem sap proteomes of wild‐type and *bhlh121* mutant plants grown in iron‐sufficient and iron‐deficient conditions. (A) Principal component analysis for the three replicates of phloem sap proteomes of wild‐type and *bhlh121* mutant plants grown in iron‐sufficient (i.e., +Fe WT and + Fe *bhlh121*) and iron‐deficient conditions (i.e., −Fe WT and −Fe *bhlh121*). (B) Heat map representation of the three replicates for phloem sap proteins wild‐type and *bhlh121* mutant plants grown under iron‐replete (i.e., +Fe WT and + Fe *bhlh121*) and iron‐deficient conditions (i.e., −Fe WT and −Fe *bhlh121*). The heat map was generated at the following URL: https://analyst‐suite.monash‐proteomics.cloud.edu.au/apps/lfq‐analyst/. (C) Repartition of the cellular location of proteins found in (+Fe WT) and within the cluster 2 of the heat map representation. Analyses were done at the following URL: https://suba.Live/toolbox.html. (D) GO term enrichment analysis for the proteins present within the cluster 2 of the heat map representation. Analyses were done using the PANTHER gene ontology platform at the following URL: http://Go.pantherdb.org.

We then compared the proteome of WT and *bhlh121* plants grown in control and iron‐deficient conditions (i.e., (+Fe WT), (−Fe WT), (+Fe *bhlh121*), and (−Fe *bhlh121*)). Here, we aimed at identifying proteins whose abundance is decreased in both iron deficiency and the *bhlh121* mutant background, as such proteins might be involved in the repression of iron uptake under iron‐replete conditions in a bHLH121‐dependent manner. Principal component analysis (PCA) was first used to evaluate the proximity of the different samples (Figure 4A). As expected, the analysis revealed that samples could be separated according to their genotypes (PC2, 27.5%). Surprisingly, the analysis also revealed that −Fe WT samples exhibited a higher level of similarity with the (+Fe *bhlh121*) ones than the others (PC1, 33.8%). This observation indicated that *bhlh121* mutants grown under iron‐sufficient conditions display iron deficiency responses, which is in agreement with the iron deficiency hypersensitivity phenotype reported for this mutant (Gao, Robe, Bettembourg, et al. 2020). It also indicates that in response to iron deficiency, *bhlh121* mutants display a specific response that most probably reflects the exacerbated effect of this stress on this genotype. We then analyzed the accumulation profile of proteins showing quantitative variations between the four types of samples. Surprisingly, none of the proteins was displaying the searched pattern of accumulation. We then concentrated our investigation on proteins that displayed qualitative variations in WT and the *bhlh121* mutant in response to iron deficiency (Data S7) and identified a cluster of 150 proteins (cluster 2) whose accumulation was higher in (+Fe WT) than in the three other conditions (Figure 4B). In terms of subcellular location, this cluster does not differ from the total phloem proteome except for a slight increase in cytosolic protein enrichment (Figure 4C). GO term enrichment analysis revealed that acetyl‐transferase activity was enriched more than 100% because three of the five pyruvate dehydrogenase subunits were enriched in this cluster. As observed previously, the translation mechanism and more precisely chaperones and ribosomal proteins were also enriched in this cluster.

### 3.5 Identification of Direct Targets of bHLH121 That Encode Proteins Identified in the Phloem Sap

Several direct targets of bHLH121 have been identified, notably genes encoding proteins involved in the regulation of iron homeostasis (i.e., bHLH transcription factors, hemerythrin domain containing E3‐ubiquitin ligases, IMAs; Kim et al. 2019; Gao, Robe, Bettembourg, et al. 2020; Gao, Robe, and Dubos 2020; Lei et al. 2020). Nonetheless, ChIP‐Seq experiments suggest that bHLH121 might regulate the expression of a larger set of genes (Kim et al. 2019). We therefore compared the list of cluster 2 that contains the 150 proteins whose accumulation is decreased in the *bhlh121* mutant and in response to iron deficiency with the bHLH121 ChIP‐Seq dataset from (Kim et al. 2019).

**FIGURE 5:**
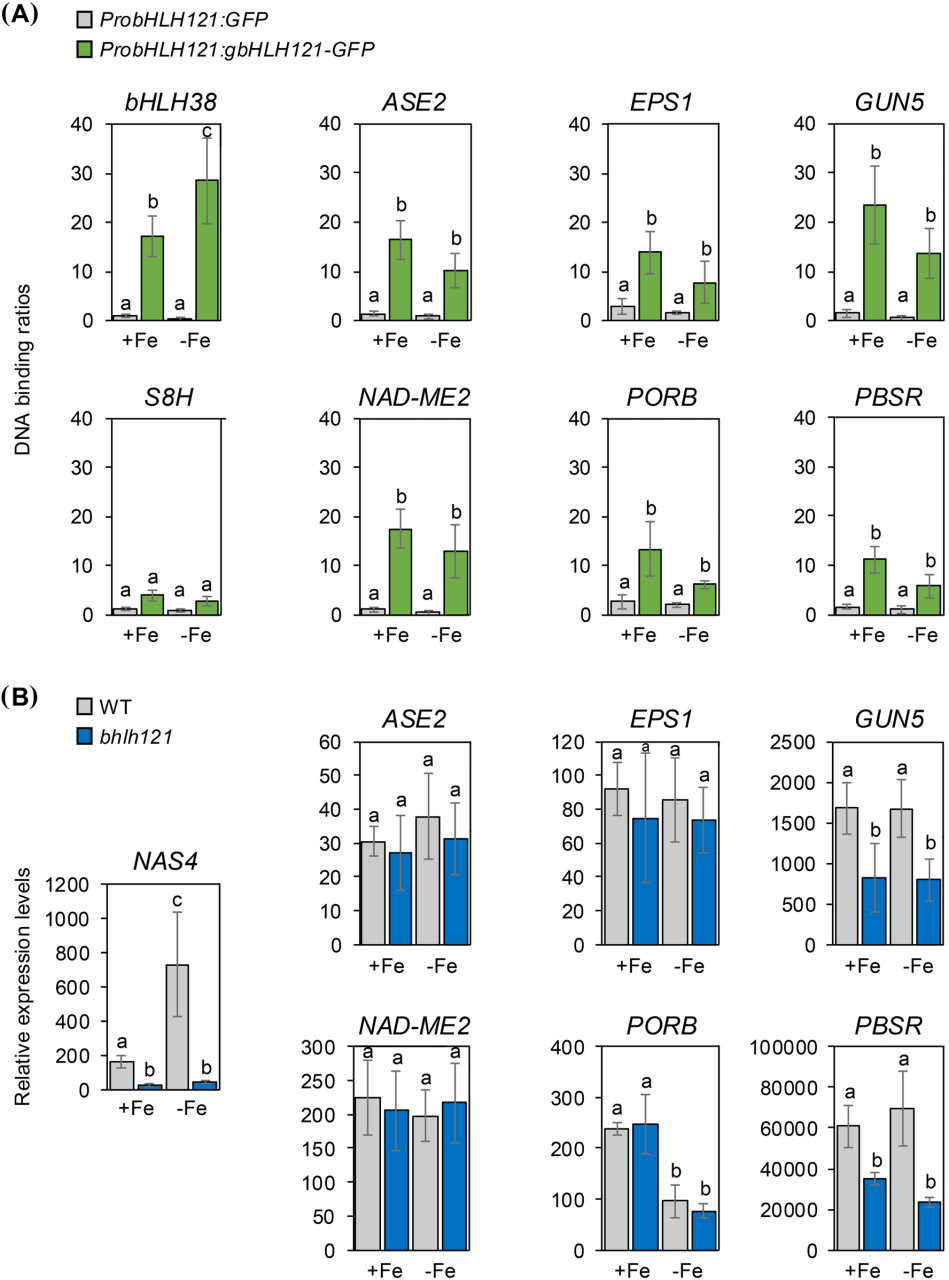
Identification of bHLH121 direct target genes that encode proteins identified in phloem sap. (A) Relative expression of *bHLH38*, *S8H*, *ASE2*, *EPS1*, *GUN5*, *NAD‐ME2*, *PORB*, and *PBSR*. Relative expression was determined by RT‐qPCR in Arabidopsis plants transferred on iron‐sufficient or iron‐deficient medium. Means within each condition with the same letter are not significantly different according to one‐way ANOVA followed by post hoc Tukey test, *p* < 0.05. Error bars show ±SD. (B) ChIP‐qPCR analysis of the binding of bHLH121 to the promoters of NAS4, *ASE2*, *EPS1*, *GUN5*, *NAD‐ME2*, *PORB*, and *PBSR*. Chromatin from the complemented *bhlh121‐2* lines expressing the *ProbHLH121:gbHLH121:GFP* construct subjected to Fe deficiency was extracted using anti‐GFP antibodies. Seedlings expressing GFP under the control of the *bHLH121* promoter (*ProbHLH121:GFP*) were used as a negative control. qPCR was used to quantify enrichment of bHLH121 on the selected gene promoters. Means within each condition with the same letter are not significantly different according to one‐way ANOVA followed by post hoc Tukey test, *p* < 0.05. Error bars show ±SD.

The comparison yielded four candidates common to both lists, namely ASE2 (GLUTAMINE PHOSPHORIBOSYL PYROPHOSPHATE AMIDOTRANSFERASE 2), EPS1 (EPSIN 1, a clathrin adaptor), NAD‐ME2 (NAD‐DEPENDENT MALIC ENZYME 2), and PSBR (PHOTOSYSTEM II SUBUNIT R), among which two were chloroplastic proteins. In order to validate these potential direct interactions between bHLH121 and the promoter of the genes encoding these four proteins, ChIP‐qPCR experiments were set up. For this purpose, *bhlh121* loss‐of‐function mutant lines complemented with the *ProbHLH121:gbHLH121‐GFP* transgene were grown together with WT lines expressing the *ProbHLH121:GFP* construct used as a negative control, in the presence or absence of iron (Gao, Robe, Bettembourg, et al. 2020). We also included in the analysis two genes whose encoded proteins were in the selected cluster, namely GUN5 (GENOMES UNCOUPLED 5) and PORB (PROTOCHLOROPHYLLIDE OXIDOREDUCTASE B). These two plastidial proteins were chosen since they are involved in the biosynthesis of one of the chlorophyll precursors, the tetrapyrrole (Tanaka et al. 2011), and are thought to be part of the plastid to nucleus iron status retrograde signaling pathway (Tran et al. 2023). Moreover, GUN5 accumulation was observed in the leaves' proteome (Data S5). *bHLH38* and *S8H* were also added to the analysis as positive and negative controls, respectively (Gao, Robe, Bettembourg, et al. 2020). We first confirm that bHLH121 directly binds to the promoter of *bHLH38* and not to the promoter of *S8H*, whether the plants were grown in the presence or in the absence of iron (Figure 5A). ChIP‐qPCR experiments revealed that bHLH121 was binding to the promoter of all six genes tested, in both iron‐replete and iron‐deficient conditions.

We then assessed the expression level by RT‐qPCR of the selected genes in WT and *bhlh121* mutant plants grown under control and iron‐deficient conditions (Figure 5B). In this experiment, *NAS4* (*NICOTIANAMINE SYNTHASE 4*) was used as control since it is a direct target of bHLH121 and since its expression is induced in response to iron deficiency (Gao, Robe, Bettembourg, et al. 2020). NAS4 is involved in the biosynthesis of nicotianamine that plays a key role in phloem sap to transport iron to the sink organs. As expected, *NAS4* expression was induced in response to iron deficiency in the WT, and not in *bhlh121*. In contrast, no variation was observed between the different genotypes and conditions tested for *ASE2*, *EPS1*, and *NAD‐ME2*. *GUN5* and *PSBR* transcript accumulation was lower in *bhlh121* than in the WT in both iron‐deficient and ‐sufficient conditions. Conversely, PORB mRNA level was reduced in response to iron deficiency in both the WT and the *bhlh121* mutant. Taken together, these observations suggest that additional bHLH transcription factors act redundantly with bHLH121 to directly regulate their expression, which is in agreement with recent studies (Gao et al. 2024).

Surprisingly, all the genes tested were expressed in the *bhlh121* mutant and under iron deficiency, indicating that some posttranscriptional or posttranslational mechanisms are at play to regulate the accumulation of the encoded proteins in the phloem sap.

## 4 Discussion

### 4.1 Arabidopsis Phloem Sap Protein Composition

We identified 1414 proteins in the phloem sap of WT Arabidopsis plants grown in iron‐repleteconditions which corresponds to two to three times more proteins identified than in previous Arabidopsis studies, and is most likely to the sensitivity progress made with recent mass spectrometers (Batailler et al. 2012; Carella et al. 2016). The size of the identified proteins as well as their associated function is in agreement with previous observations made for Arabidopsis samples but also for samples from different plant species such as rapeseed ( *Brassica napus* ) and cucumber ( *Cucumis sativus* ; Liu et al. 2021) (Figure 1). For instance, the GO term enrichment analysis we conducted revealed the presence of proteins related to “metabolism,” notably associated with the glycolysis pathway, as described in (Giavalisco et al. 2006). We also confirmed the presence of ribosomal proteins, even if proofs supporting a translational activity in sieve elements remains elusive. Similar observations were made with proteins related to “stress responses.”

### 4.2 Phloem Sap Protein Composition is Deeply Modified in Response to Iron Availability

How iron availability affects gene expressions at the posttranscriptional level has been the focus of several studies where proteomic approaches were used (Mai et al. 2015; Pan et al. 2015; Zargar et al. 2015). Such studies highlighted, for instance, that the accumulation of proteins whose function was related to the maintenance of iron homeostasis depended on the amount of iron that was present in the growth media. This is for instance the case for IRT1 and FRO2 that are involved in the uptake of iron by the plant root, but also for F6ʹH1 (FERULOYL CoA 6ʹ‐HYDROXYLASE 1), S8H (SCOPOLETIN 8‐HYDROXYLASE), and BGLU42 (β‐GLUCOSIDASE 42) that are involved in the biosynthesis and secretion of coumarins into the rhizosphere, a process that facilitates the uptake of iron, or FER1, 3, and 4 that are involved in the transient storage of iron. Additional proteins using iron as a cofactor were also among the identified proteins (e.g., PORB or At‐NEET). The accumulation of OPT3, whose activity is necessary for the long‐distance signaling of leaves' iron status to the roots, via the phloem sap, to modulate the activity of the iron uptake machinery, is also affected by the availability of iron. Despite the importance of this signaling process in plant iron nutrition and the maintenance of iron homeostasis, few studies have investigated how iron availability influences the phloem sap proteome.

We observed that the accumulation of about half of the total phloem sap protein pool is affected in response to fluctuation in iron availability (i.e., iron‐replete vs. iron‐deficient growth conditions). This observation indicates that there is a deep modification of the phloem sap protein composition that might reflect iron deficiency‐dependent changes known to occur at the whole plant level (e.g., chlorosis, growth reduction). Interestingly, most proteins whose accumulation is affected by the iron deficiency treatment in the phloem sap were not affected at the whole leave proteome level, suggesting that (i) the identified proteins are either specific or overrepresented in phloem sap and that (ii) there is a phloem sap‐specific response to iron deficiency. If, as above‐stated, no evidence supports that proteins are directly translated in the phloem sap, it cannot be excluded that proteins could be transcribed and/or translated in an iron availability‐dependent manner in companion cells, thanks to specific sets of ribosomal proteins and other elements implicated in translation, like translation initiation factors. For instance, we have found 42 ribosomal proteins displaying a decreased accumulation in response to iron deficiency whose function has to be elucidated.

Surprisingly, among the identified proteins, IMAs were not found, neither in the control nor in the iron‐deficient conditions. Whether or not the amount of this small protein in the phloem sap is too low to be detected is an open question. Nonetheless, if the implication of IMAs in long‐distance signaling of leaves’ iron status to the roots has been clearly demonstrated (Grillet et al. 2018; Hirayama et al. 2018), its localization in the phloem sap is still to be proven. For instance, it was recently shown that the YFP‐IMA1 fusion protein localization is restricted around the vasculature when expressed under its native promoter in plants grown in iron‐replete conditions. The YFP‐IMA1 fusion protein localization extends to the pericycle, the endodermis, and the cortex tissues in response to iron deficiency (Cao et al. 2024). YFP‐IMA1 localization around the vasculature suggests that the fusion protein accumulates in phloem companion cells, but this assertion remains to be formally confirmed (Grillet et al. 2018; Hirayama et al. 2018). Similarly, HY5 was not among the identified proteins in the phloem sap, even in iron‐deficient conditions. This later observation is likely due to the weak detection level of lowly expressed proteins such as transcription factors.

In contrast, we found TOPLESS RELATED 1 (TPR1), a transcriptional repressor, among the proteins whose accumulation is increased in the phloem sap of WT plants subjected to iron deficiency (Li et al. 2022). It is proposed that under iron‐sufficient conditions, TRP1 interaction with bHLH11 (whose expression is induced in this condition) inhibits the activity of clade IVc bHLH transcription factors and, therefore, the expression of clade Ib bHLH and their direct target (e.g., *IRT1*) to limit iron uptake and avoid iron toxicity (Li et al. 2022). Determining whether or not this apparent negative correlation between the expression of *bHLH11* and the accumulation of TPR1 in the phloem sap is part of the long‐distance signaling mechanism that regulates iron homeostasis in plants might be an appealing hypothesis that deserves further investigation in future studies.

Long‐distance signaling of leaf nutritional status was also shown to include proteins involved in the modification of the redox status. This is, for instance, the case for the systemic regulation of nitrogen acquisition that requires the CC‐type glutaredoxin CEP DOWNSTREAM 1 and 2 (CEPD1/ROXY6 and CEPD2/ROXY9; Ohkubo et al. 2017). Interestingly, different glutathione S‐transferases and thioredoxins displayed specific accumulation either in iron‐sufficient or iron‐deficient conditions.

Since it was previously shown that iron excess leads to a reversible phloem‐specific increase of callose deposition associated with a decrease of plasmodesmatal permeability (O'Lexy et al. 2018), one might hypothesize that such mechanism might participate in the modulation of long‐distance signaling of leaves’ iron status to the roots. Whether the accumulation of specific β‐glucanases implicated in callose degradation in response to iron deficiency participates in this process is also an appealing hypothesis that deserves to be tested.

### 4.3 Phloem Sap Protein Composition Relies on bHLH121 Activity

Phloem sap protein composition is deeply affected by the availability of iron. We have, for instance, observed profound modifications at both protein identity and protein quantification levels between phloem sap from plants grown under iron‐replete and iron‐deficient conditions. These variations in phloem sap protein composition reflect changes observed at the whole plant level (e.g., leaf chlorosis). Most strikingly, we observed that phloem sap of *bhlh121* loss‐of‐function mutant grown under iron‐replete conditions was closely related to that of WT plants grown under iron deficiency. It is noteworthy that under iron‐replete conditions, bHLH121 mostly accumulates in cells localized near the vasculature (Gao, Robe, Bettembourg, et al. 2020). Such localization is in adequacy with the potential role of bHLH121 in modulating phloem sap protein compositions and suggests that bHLH121 might play a pivotal role in this process. In support of this assertion, among the six genes we confirmed as direct targets of bHLH121, five encode proteins with a molecular weight that is compatible with an entrance into the sieve elements via plasmodesmata (i.e., ASE2, EPS1, NAD‐ME2, PORB, and PSBR with a predicted molecular weight comprised between 14.5 and 66 kDa). Interestingly, among these six bHLH121 direct targets, two encode proteins involved in the plastid‐to‐nucleus retrograde signaling pathway (i.e., GUN5 and PORB; Mochizuki et al. 2001; Dong et al. 2007), a mechanism known to participate in the maintenance of iron homeostasis (Salome et al. 2013; Tissot et al. 2014; Balparda et al. 2021). Nonetheless, what would be the role, if any of these two enzymes involved in the biosynthesis of chlorophylls in the long‐distance signaling process of leaves’ iron status to the roots is unclear. If this study revealed that the phloem sap proteome is affected by bHLH121 activity, expression analysis highlighted that the expression of the assayed genes also depended on other regulatory proteins likely belonging to the bHLH clade IVc (Gao and Dubos 2024). This comparison also supports that it is most likely that some posttranscriptional or posttranslational regulatory mechanisms are at play to modulate the phloem sap protein composition in response to iron availability.

## Author Contributions

B.N. and D.C. conceived and designed the experiments. B.N., K.M., G.F., and R.V. performed the experiments. B.N., K.M., G.F., R.V., D.V., S.V., and D.C. analyzed the data. D.C. contributed reagents/materials/analysis tools. R.V. and S.V. provided access to mass spectrometry facilities. B.N. and D.C. wrote the paper with the help of all the authors.

## Supporting information


**Data S1.** List of proteins identified in the phloem sap of wild type plants grown in iron‐sufficient conditions (+Fe WT).
**Data S2.** GO term enrichment analysis for the proteins identified in the phloem sap of wild type plants grown in iron‐sufficient conditions (+Fe WT).
**Data S3.** List of proteins differentially accumulated in the phloem sap of wild type and *bhlh121* mutant grown in iron sufficiency or subjected to iron deficiency.
**Data S4.** List of ribosomal proteins present in the (+Fe WT) phloem sap whose accumulation is decreased in −Fe WT.
**Data S5.** List of proteins identified in leaves and differential accumulation analysis in response to iron availability.
**Data S6.** bHLH‐related cis element localization in the genomic DNA sequence of the putative *bHLH121* targets.
**Data S7.** List of phloem sap proteins qualitatively varying between WT and *bhlh121*, and + Fe and −Fe.
**Table S1.** List of primers used in this study.

## Data Availability

All raw mass spectrometry data and Maxquant files generated have been deposited to the ProteomeXchange Consortium via the PRIDE partner repository with the data set identifier PXD061925.

## References

[ppl70336-bib-0001] Balk, J. , and M. Pilon . 2011. “Ancient and Essential: The Assembly of Iron‐Sulfur Clusters in Plants.” Trends in Plant Science 16, no. 4: 218–226. 21257336 10.1016/j.tplants.2010.12.006

[ppl70336-bib-0002] Balparda, M. , A. M. Armas , D. F. Gomez‐Casati , and M. A. Pagani . 2021. “PAP/SAL1 Retrograde Signaling Pathway Modulates Iron Deficiency Response in Alkaline Soils.” Plant Science 304: 110808. 33568304 10.1016/j.plantsci.2020.110808

[ppl70336-bib-0003] Batailler, B. , T. Lemaitre , F. Vilaine , et al. 2012. “Soluble and Filamentous Proteins in Arabidopsis Sieve Elements.” Plant, Cell & Environment 35, no. 7: 1258–1273. 10.1111/j.1365-3040.2012.02487.x 22292537

[ppl70336-bib-0004] Berger, N. , F. Vignols , J. Przybyla‐Toscano , et al. 2020. “Identification of Client Iron‐Sulfur Proteins of the Chloroplastic NFU2 Transfer Protein in *Arabidopsis thaliana* .” Journal of Experimental Botany 71, no. 14: 4171–4187. 32240305 10.1093/jxb/eraa166

[ppl70336-bib-0005] Briat, J. F. , C. Dubos , and F. Gaymard . 2015. “Iron Nutrition, Biomass Production, and Plant Product Quality.” Trends in Plant Science 20, no. 1: 33–40. 25153038 10.1016/j.tplants.2014.07.005

[ppl70336-bib-0006] Cao, M. , M. P. Platre , H. H. Tsai , et al. 2024. “Spatial IMA1 Regulation Restricts Root Iron Acquisition on MAMP Perception.” Nature 625, no. 7996: 750–759. 38200311 10.1038/s41586-023-06891-y PMC11181898

[ppl70336-bib-0007] Carella, P. , J. Merl‐Pham , D. C. Wilson , et al. 2016. “Comparative Proteomics Analysis of Phloem Exudates Collected During the Induction of Systemic Acquired Resistance.” Plant Physiology 171, no. 2: 1495–1510. 27208255 10.1104/pp.16.00269 PMC4902610

[ppl70336-bib-0008] Castaneda, V. , E. M. Gonzalez , and S. Wienkoop . 2021. “Phloem Sap Proteins Are Part of a Core Stress Responsive Proteome Involved in Drought Stress Adjustment.” Frontiers in Plant Science 12: 625224. 33603764 10.3389/fpls.2021.625224 PMC7884324

[ppl70336-bib-0009] Chen, J. , and W. R. Browne . 2018. “Photochemistry of Iron Complexes.” Coordination Chemistry Reviews 374: 15–35.

[ppl70336-bib-0010] Chia, J. C. , J. Yan , M. Rahmati Ishka , et al. 2023. “Loss of OPT3 Function Decreases Phloem Copper Levels and Impairs Crosstalk Between Copper and Iron Homeostasis and Shoot‐To‐Root Signaling in *Arabidopsis thaliana* .” Plant Cell 35, no. 6: 2157–2185. 36814393 10.1093/plcell/koad053 PMC10226573

[ppl70336-bib-0011] Colangelo, E. P. , and M. L. Guerinot . 2004. “The Essential Basic Helix‐Loop‐Helix Protein FIT1 Is Required for the Iron Deficiency Response.” Plant Cell 16, no. 12: 3400–3412. 15539473 10.1105/tpc.104.024315 PMC535881

[ppl70336-bib-0012] Connolly, E. L. , and M. Guerinot . 2002. “Iron Stress in Plants.” Genome Biology 3, no. 8: 1–4. 10.1186/gb-2002-3-8-reviews1024 PMC139400 12186653

[ppl70336-bib-0013] Corbesier, L. , C. Vincent , S. Jang , et al. 2007. “FT Protein Movement Contributes to Long‐Distance Signaling in Floral Induction of Arabidopsis.” Science 316, no. 5827: 1030–1033. 17446353 10.1126/science.1141752

[ppl70336-bib-0014] Cox, J. , and M. Mann . 2008. “MaxQuant Enables High Peptide Identification Rates, Individualized ppb‐Range Mass Accuracies and Proteome‐Wide Protein Quantification.” Nature Biotechnology 26, no. 12: 1367–1372. 10.1038/nbt.1511 19029910

[ppl70336-bib-0015] Cox, J. , N. Neuhauser , A. Michalski , R. A. Scheltema , J. V. Olsen , and M. Mann . 2011. “Andromeda: A Peptide Search Engine Integrated Into the MaxQuant Environment.” Journal of Proteome Research 10, no. 4: 1794–1805. 21254760 10.1021/pr101065j

[ppl70336-bib-0016] Czechowski, T. , M. Stitt , T. Altmann , M. K. Udvardi , and W. R. Scheible . 2005. “Genome‐Wide Identification and Testing of Superior Reference Genes for Transcript Normalization in Arabidopsis.” Plant Physiology 139, no. 1: 5–17. 16166256 10.1104/pp.105.063743 PMC1203353

[ppl70336-bib-0017] Dong, H. , Y. Deng , J. Mu , et al. 2007. “The Arabidopsis Spontaneous Cell Death1 Gene, Encoding a Zeta‐Carotene Desaturase Essential for Carotenoid Biosynthesis, Is Involved in Chloroplast Development, Photoprotection and Retrograde Signalling.” Cell Research 17, no. 5: 458–470. 17468780 10.1038/cr.2007.37

[ppl70336-bib-0018] Fourcroy, P. , P. Siso‐Terraza , D. Sudre , et al. 2014. “Involvement of the ABCG37 Transporter in Secretion of Scopoletin and Derivatives by Arabidopsis Roots in Response to Iron Deficiency.” New Phytologist 201, no. 1: 155–167. 24015802 10.1111/nph.12471

[ppl70336-bib-0019] Gao, F. , and C. Dubos . 2021. “Transcriptional Integration of Plant Responses to Iron Availability.” Journal of Experimental Botany 72, no. 6: 2056–2070. 33246334 10.1093/jxb/eraa556

[ppl70336-bib-0020] Gao, F. , and C. Dubos . 2023. “Chromatin Immunoprecipitation (ChIP) to Study the Transcriptional Regulatory Network That Controls Iron Homeostasis in *Arabidopsis thaliana* .” Methods in Molecular Biology 2665: 85–94. 37166595 10.1007/978-1-0716-3183-6_8

[ppl70336-bib-0021] Gao, F. , and C. Dubos . 2024. “The Arabidopsis bHLH Transcription Factor Family.” Trends in Plant Science 29, no. 6: 668–680. 38143207 10.1016/j.tplants.2023.11.022

[ppl70336-bib-0022] Gao, F. , M. Li , and C. Dubos . 2024. “bHLH121 and Clade IVc bHLH Transcription Factors Synergistically Function to Regulate Iron Homeostasis in *Arabidopsis thaliana* .” Journal of Experimental Botany 75, no. 10: 2933–2950. 38441949 10.1093/jxb/erae072

[ppl70336-bib-0023] Gao, F. , K. Robe , M. Bettembourg , et al. 2020. “The Transcription Factor bHLH121 Interacts With bHLH105 (ILR3) and Its Closest Homologs to Regulate Iron Homeostasis in Arabidopsis.” Plant Cell 32, no. 2: 508–524. 31776233 10.1105/tpc.19.00541 PMC7008485

[ppl70336-bib-0024] Gao, F. , K. Robe , and C. Dubos . 2020. “Further Insights Into the Role of bHLH121 in the Regulation of Iron Homeostasis in *Arabidopsis thaliana* .” Plant Signaling & Behavior 15, no. 10: 1795582. 32692954 10.1080/15592324.2020.1795582 PMC8550535

[ppl70336-bib-0025] Giavalisco, P. , K. Kapitza , A. Kolasa , A. Buhtz , and J. Kehr . 2006. “Towards the Proteome of *Brassica napus* Phloem Sap.” Proteomics 6, no. 3: 896–909. 16400686 10.1002/pmic.200500155

[ppl70336-bib-0026] Grillet, L. , P. Lan , W. Li , G. Mokkapati , and W. Schmidt . 2018. “Iron man Is a Ubiquitous Family of Peptides That Control Iron Transport in Plants.” Nature Plants 4, no. 11: 953–963. 30323182 10.1038/s41477-018-0266-y

[ppl70336-bib-0027] Grusak, M. A. , and S. Pezeshgi . 1996. “Shoot‐To‐Root Signal Transmission Regulates Root Fe(III) Reductase Activity in the Dgl Mutant of Pea.” Plant Physiology 110, no. 1: 329–334. 12226184 10.1104/pp.110.1.329 PMC157724

[ppl70336-bib-0028] Hirayama, T. , G. J. Lei , N. Yamaji , N. Nakagawa , and J. F. Ma . 2018. “The Putative Peptide Gene FEP1 Regulates Iron Deficiency Response in Arabidopsis.” Plant & Cell Physiology 59, no. 9: 1739–1752. 30032190 10.1093/pcp/pcy145

[ppl70336-bib-0029] Jakoby, M. , H. Y. Wang , W. Reidt , B. Weisshaar , and P. Bauer . 2004. “FRU (BHLH029) is Required for Induction of Iron Mobilization Genes in *Arabidopsis thaliana* .” FEBS Letters 577, no. 3: 528–534. 15556641 10.1016/j.febslet.2004.10.062

[ppl70336-bib-0030] Kim, S. A. , I. S. LaCroix , S. A. Gerber , and M. L. Guerinot . 2019. “The Iron Deficiency Response in *Arabidopsis thaliana* Requires the Phosphorylated Transcription Factor URI.” Proceedings of the National Academy of Sciences of the United States of America 116, no. 50: 24933–24942. 31776249 10.1073/pnas.1916892116 PMC6911256

[ppl70336-bib-0031] Laemmli, U. K. 1970. “Cleavage of Structural Proteins During the Assembly of the Head of Bacteriophage T4.” Nature 227, no. 5259: 680–685. 5432063 10.1038/227680a0

[ppl70336-bib-0032] Lei, R. , Y. Li , Y. Cai , et al. 2020. “bHLH121 Functions as a Direct Link That Facilitates the Activation of FIT by bHLH IVc Transcription Factors for Maintaining Fe Homeostasis in Arabidopsis.” Molecular Plant 13, no. 4: 634–649. 31962167 10.1016/j.molp.2020.01.006

[ppl70336-bib-0033] Li, M. , S. Watanabe , F. Gao , and C. Dubos . 2023. “Iron Nutrition in Plants: Towards a New Paradigm?” Plants 12, no. 2: 384. 36679097 10.3390/plants12020384 PMC9862363

[ppl70336-bib-0034] Li, Y. , R. Lei , M. Pu , et al. 2022. “bHLH11 Inhibits bHLH IVc Proteins by Recruiting the Topless/Topless‐Related Corepressors.” Plant Physiology 188, no. 2: 1335–1349. 34894263 10.1093/plphys/kiab540 PMC8825326

[ppl70336-bib-0035] Li, Y. , C. K. Lu , C. Y. Li , et al. 2021. “Iron Man Interacts With Brutus to Maintain Iron Homeostasis in Arabidopsis.” Proceedings of the National Academy of Sciences of the United States of America 118, no. 39: e2109063118. 34548401 10.1073/pnas.2109063118 PMC8488653

[ppl70336-bib-0036] Liang, G. 2022. “Iron Uptake, Signaling, and Sensing in Plants.” Plant Communications 3, no. 5: 100349. 35706354 10.1016/j.xplc.2022.100349 PMC9483112

[ppl70336-bib-0037] Lin, M. K. , H. Belanger , Y. J. Lee , et al. 2007. “Flowering Locus T Protein May Act as the Long‐Distance Florigenic Signal in the Cucurbits.” Plant Cell 19, no. 5: 1488–1506. 17540715 10.1105/tpc.107.051920 PMC1913722

[ppl70336-bib-0038] Liu, Y. , T. Lin , M. V. Valencia , C. Zhang , and Z. Lv . 2021. “Unraveling the Roles of Vascular Proteins Using Proteomics.” Molecules 26, no. 3: 667. 33514014 10.3390/molecules26030667 PMC7865979

[ppl70336-bib-0039] Mai, H. J. , C. Lindermayr , C. von Toerne , C. Fink‐Straube , J. Durner , and P. Bauer . 2015. “Iron and FER‐Like Iron Deficiency‐Induced Transcription Factor‐Dependent Regulation of Proteins and Genes in *Arabidopsis thaliana* Roots.” Proteomics 15, no. 17: 3030–3047. 25951126 10.1002/pmic.201400351

[ppl70336-bib-0040] Mankotia, S. , A. Dubey , P. Jakhar , et al. 2025. “Elongated Hypocotyl 5 (HY5) and Popeye (PYE) Regulate Intercellular Iron Transport in Plants.” Plant, Cell & Environment 48, no. 4: 2647–2661. 10.1111/pce.15090 39136421

[ppl70336-bib-0041] Mankotia, S. , P. Jakhar , and S. B. Satbhai . 2024. “HY5: A Key Regulator for Light‐Mediated Nutrient Uptake and Utilization by Plants.” New Phytologist 241, no. 5: 1929–1935. 38178773 10.1111/nph.19516

[ppl70336-bib-0042] Mochizuki, N. , J. A. Brusslan , R. Larkin , A. Nagatani , and J. Chory . 2001. “Arabidopsis Genomes Uncoupled 5 (GUN5) Mutant Reveals the Involvement of mg‐Chelatase H Subunit in Plastid‐To‐Nucleus Signal Transduction.” Proceedings of the National Academy of Sciences of the United States of America 98, no. 4: 2053–2058. 11172074 10.1073/pnas.98.4.2053 PMC29380

[ppl70336-bib-0043] Ohkubo, Y. , M. Tanaka , R. Tabata , M. Ogawa‐Ohnishi , and Y. Matsubayashi . 2017. “Shoot‐To‐Root Mobile Polypeptides Involved in Systemic Regulation of Nitrogen Acquisition.” Nature Plants 3: 17029. 28319056 10.1038/nplants.2017.29

[ppl70336-bib-0044] O'Lexy, R. , K. Kasai , N. Clark , T. Fujiwara , R. Sozzani , and K. L. Gallagher . 2018. “Exposure to Heavy Metal Stress Triggers Changes in Plasmodesmatal Permeability via Deposition and Breakdown of Callose.” Journal of Experimental Botany 69, no. 15: 3715–3728. 29901781 10.1093/jxb/ery171 PMC6022669

[ppl70336-bib-0045] Paffrath, V. , Y. A. Tandron Moya , G. Weber , N. von Wirén , and R. F. H. Giehl . 2024. “A Major Role of Coumarin‐Dependent Ferric Iron Reduction in Strategy I‐Type Iron Acquisition in Arabidopsis.” Plant Cell 36, no. 3: 642–664. 38016103 10.1093/plcell/koad279 PMC10896297

[ppl70336-bib-0046] Pan, I. C. , H. H. Tsai , Y. T. Cheng , T. N. Wen , T. J. Buckhout , and W. Schmidt . 2015. “Post‐Transcriptional Coordination of the Arabidopsis Iron Deficiency Response Is Partially Dependent on the E3 Ligases Ring Domain Ligase1 (RGLG1) and Ring Domain Ligase2 (RGLG2).” Molecular & Cellular Proteomics 14, no. 10: 2733–2752. 26253232 10.1074/mcp.M115.048520 PMC4597148

[ppl70336-bib-0047] Rajniak, J. , R. F. H. Giehl , E. Chang , I. Murgia , N. von Wirén , and E. S. Sattely . 2018. “Biosynthesis of Redox‐Active Metabolites in Response to Iron Deficiency in Plants.” Nature Chemical Biology 14, no. 5: 442–450. 29581584 10.1038/s41589-018-0019-2 PMC6693505

[ppl70336-bib-0048] Robe, K. , G. Conejero , F. Gao , et al. 2021. “Coumarin Accumulation and Trafficking in *Arabidopsis thaliana* : A Complex and Dynamic Process.” New Phytologist 229, no. 4: 2062–2079. 33205512 10.1111/nph.17090

[ppl70336-bib-0049] Robe, K. , F. Gao , P. Bonillo , et al. 2020. “Sulphur Availability Modulates *Arabidopsis thaliana* Responses to Iron Deficiency.” PLoS One 15, no. 8: e0237998. 32817691 10.1371/journal.pone.0237998 PMC7440645

[ppl70336-bib-0050] Robe, K. , E. Izquierdo , F. Vignols , H. Rouached , and C. Dubos . 2021. “The Coumarins: Secondary Metabolites Playing a Primary Role in Plant Nutrition and Health.” Trends in Plant Science 26, no. 3: 248–259. 33246890 10.1016/j.tplants.2020.10.008

[ppl70336-bib-0051] Robinson, N. J. , C. M. Procter , E. L. Connolly , and M. L. Guerinot . 1999. “A Ferric‐Chelate Reductase for Iron Uptake From Soils.” Nature 397, no. 6721: 694–697. 10067892 10.1038/17800

[ppl70336-bib-0052] Rogers, E. E. , and M. L. Guerinot . 2002. “FRD3, a Member of the Multidrug and Toxin Efflux Family, Controls Iron Deficiency Responses in Arabidopsis.” Plant Cell 14, no. 8: 1787–1799. 12172022 10.1105/tpc.001495 PMC151465

[ppl70336-bib-0053] Salome, P. A. , M. Oliva , D. Weigel , and U. Kramer . 2013. “Circadian Clock Adjustment to Plant Iron Status Depends on Chloroplast and Phytochrome Function.” EMBO Journal 32, no. 4: 511–523. 23241948 10.1038/emboj.2012.330 PMC3579136

[ppl70336-bib-0054] Santi, S. , and W. Schmidt . 2009. “Dissecting Iron Deficiency‐Induced Proton Extrusion in Arabidopsis Roots.” New Phytologist 183, no. 4: 1072–1084. 19549134 10.1111/j.1469-8137.2009.02908.x

[ppl70336-bib-0055] Schmid, N. B. , R. F. Giehl , S. Döll , et al. 2014. “Feruloyl‐CoA 6'‐Hydroxylase1‐Dependent Coumarins Mediate Iron Acquisition From Alkaline Substrates in Arabidopsis.” Plant Physiology 164, no. 1: 160–172. 24246380 10.1104/pp.113.228544 PMC3875798

[ppl70336-bib-0056] Selote, D. , R. Samira , A. Matthiadis , J. W. Gillikin , and T. A. Long . 2015. “Iron‐Binding E3 Ligase Mediates Iron Response in Plants by Targeting Basic Helix‐Loop‐Helix Transcription Factors.” Plant Physiology 167, no. 1: 273–286. 25452667 10.1104/pp.114.250837 PMC4281009

[ppl70336-bib-0057] Shah, A. D. , R. J. A. Goode , C. Huang , D. R. Powell , and R. B. Schittenhelm . 2020. “LFQ‐Analyst: An Easy‐To‐Use Interactive Web Platform to Analyze and Visualize Label‐Free Proteomics Data Preprocessed With MaxQuant.” Journal of Proteome Research 19, no. 1: 204–211. 31657565 10.1021/acs.jproteome.9b00496

[ppl70336-bib-0058] Sisó‐Terraza, P. , A. Luis‐Villarroya , P. Fourcroy , et al. 2016. “Accumulation and Secretion of Coumarinolignans and Other Coumarins in *Arabidopsis thaliana* Roots in Response to Iron Deficiency at High pH.” Frontiers in Plant Science 23, no. 7: 1711. 10.3389/fpls.2016.01711 PMC5120119 27933069

[ppl70336-bib-0059] Siwinska, J. , K. Siatkowska , A. Olry , et al. 2018. “Scopoletin 8‐Hydroxylase: A Novel Enzyme Involved in Coumarin Biosynthesis and Iron‐Deficiency Responses in Arabidopsis.” Journal of Experimental Botany 69, no. 7: 1735–1748. 29361149 10.1093/jxb/ery005 PMC5888981

[ppl70336-bib-0060] Stacey, M. G. , A. Patel , W. E. McClain , et al. 2008. “The Arabidopsis AtOPT3 Protein Functions in Metal Homeostasis and Movement of Iron to Developing Seeds.” Plant Physiology 146, no. 2: 589–601. 18083798 10.1104/pp.107.108183 PMC2245856

[ppl70336-bib-0061] Takahashi, F. , and K. Shinozaki . 2019. “Long‐Distance Signaling in Plant Stress Response.” Current Opinion in Plant Biology 47: 106–111. 30445314 10.1016/j.pbi.2018.10.006

[ppl70336-bib-0062] Tanaka, R. , K. Kobayashi , and T. Masuda . 2011. “Tetrapyrrole Metabolism in *Arabidopsis thaliana* .” Arabidopsis Book 9: e0145. 22303270 10.1199/tab.0145 PMC3268503

[ppl70336-bib-0063] Tetyuk, O. , U. F. Benning , and S. Hoffmann‐Benning . 2013. “Collection and Analysis of Arabidopsis Phloem Exudates Using the EDTA‐Facilitated Method.” Journal of Visualized Experiments, no. 80: e51111. 24192764 10.3791/51111 PMC3960974

[ppl70336-bib-0064] Tissot, N. , J. Przybyla‐Toscano , G. Reyt , et al. 2014. “Iron Around the Clock.” Plant Science 224: 112–119. 24908512 10.1016/j.plantsci.2014.03.015

[ppl70336-bib-0065] Touraine, B. , F. Vignols , J. Przybyla‐Toscano , et al. 2019. “Iron‐Sulfur Protein NFU2 Is Required for Branched‐Chain Amino Acid Synthesis in Arabidopsis Roots.” Journal of Experimental Botany 70, no. 6: 1875–1889. 30785184 10.1093/jxb/erz050

[ppl70336-bib-0066] Tran, L. H. , J. G. Kim , and S. Jung . 2023. “Expression of the Arabidopsis mg‐Chelatase H Subunit Alleviates Iron Deficiency‐Induced Stress in Transgenic Rice.” Frontiers in Plant Science 14: 1098808. 36938029 10.3389/fpls.2023.1098808 PMC10017980

[ppl70336-bib-0067] Trofimov, K. , S. Mankotia , M. Ngigi , D. Baby , S. B. Satbhai , and P. Bauer . 2025. “Shedding Light on Iron Nutrition: Exploring Intersections of Transcription Factor Cascades in Light and Iron Deficiency Signaling.” Journal of Experimental Botany 76, no. 3: 787–802. 39115876 10.1093/jxb/erae324 PMC11805591

[ppl70336-bib-0068] Tsai, H. H. , J. Rodríguez‐Celma , P. Lan , Y. C. Wu , I. C. Vélez‐Bermúdez , and W. Schmidt . 2018. “Scopoletin 8‐Hydroxylase‐Mediated Fraxetin Production Is Crucial for Iron Mobilization.” Plant Physiology 177, no. 1: 194–207. 29559590 10.1104/pp.18.00178 PMC5933141

[ppl70336-bib-0069] Vanholme, R. , L. Sundin , K. C. Seetso , et al. 2019. “COSY Catalyses Trans‐Cis Isomerization and Lactonization in the Biosynthesis of Coumarins.” Nature Plants 5, no. 10: 1066–1075. 31501530 10.1038/s41477-019-0510-0

[ppl70336-bib-0070] Vert, G. , N. Grotz , F. Dédaldéchamp , et al. 2002. “IRT1, an Arabidopsis Transporter Essential for Iron Uptake From the Soil and for Plant Growth.” Plant Cell 14, no. 6: 1223–1233. 12084823 10.1105/tpc.001388 PMC150776

[ppl70336-bib-0071] Walz, C. , P. Giavalisco , M. Schad , M. Juenger , J. Klose , and J. Kehr . 2004. “Proteomics of Curcurbit Phloem Exudate Reveals a Network of Defence Proteins.” Phytochemistry 65, no. 12: 1795–1804. 15276438 10.1016/j.phytochem.2004.04.006

[ppl70336-bib-0072] Wang, J. , P. Lan , H. Gao , L. Zheng , W. Li , and W. Schmidt . 2013. “Expression Changes of Ribosomal Proteins in Phosphate‐ and Iron‐Deficient Arabidopsis Roots Predict Stress‐Specific Alterations in Ribosome Composition.” BMC Genomics 14: 783. 24225185 10.1186/1471-2164-14-783 PMC3830539

[ppl70336-bib-0073] Yuan, Y. X. , J. Zhang , D. W. Wang , and H. Q. Ling . 2005. “AtbHLH29 of *Arabidopsis thaliana* Is a Functional Ortholog of Tomato FER Involved in Controlling Iron Acquisition in Strategy I Plants.” Cell Research 15, no. 8: 613–621. 16117851 10.1038/sj.cr.7290331

[ppl70336-bib-0074] Zargar, S. M. , G. K. Agrawal , R. Rakwal , and Y. Fukao . 2015. “Quantitative Proteomics Reveals Role of Sugar in Decreasing Photosynthetic Activity due to Fe Deficiency.” Frontiers in Plant Science 6: 592. 26284105 10.3389/fpls.2015.00592 PMC4522737

[ppl70336-bib-0075] Zhai, Z. , S. R. Gayomba , H. I. Jung , et al. 2014. “OPT3 Is a Phloem‐Specific Iron Transporter That Is Essential for Systemic Iron Signaling and Redistribution of Iron and Cadmium in Arabidopsis.” Plant Cell 26, no. 5: 2249–2264. 24867923 10.1105/tpc.114.123737 PMC4079381

