# Supplementary material for: Impact of Iron Deficiency on the Arabidopsis thaliana Phloem Sap Proteome, a Key Role for bHLH121
Source: Physiol Plant. 2025 Jun 19;177(3):e70336. doi: 10.1111/ppl.70336 (PMC12177908; doi:10.1111/ppl.70336)
Supplement: Supplementary file 1 — Data S1. List of proteins identified in the phloem sap of wild type plants grown in iron‐sufficient conditions (+Fe WT). Data S2. GO term enrichment analysis for the proteins identified in the phloem sap of wild type plants grown in iron‐sufficient conditions (+Fe WT). Data S3. List of proteins differentially accumulated in the phloem sap of wild type and bhlh121 mutant grown in iron sufficiency or subjected to iron deficiency. Data S4. List of ribosomal proteins present in the (+Fe WT) phloem sap whose accumulation is decreased in −Fe WT. Data S5. List of proteins identified in leaves and differential accumulation analysis in response to iron availability. Data S6. bHLH‐related cis element localization in the genomic DNA sequence of the putative bHLH121 targets. Data S7. List of phloem sap proteins qualitatively varying between WT and bhlh121, and + Fe and −Fe. Table S1. List of primers used in this study. [file PPL-177-e70336-s001.docx]

**Supplemental Data Set 1:** list of proteins identified in the phloem sap of wild type plants grown in iron sufficiency conditions (+Fe WT).

**Supplemental Data Set 2:** GO term enrichment analysis for the proteins identified in the phloem sap of wild type plants grown in iron sufficiency condition (+Fe WT).

**Supplemental Data Set 3:** list of proteins differentially accumulated in the phloem sap of wild type and *bhlh121* mutant grown in iron sufficiency or subjected to iron deficiency.

**Supplemental Data Set 4:** list of ribosomal proteins present in the +Fe WT phloem sap whose accumulation is decreased in -Fe WT.

**Supplemental Data Set 5:** list of proteins identified in leaves and differential accumulation analysis in response to iron availability.

**Supplemental Data Set 6:** bHLH related cis element localization in the genomic DNA sequence of the putative bHLH121 targets.

**Supplemental Data Set 7:** lists of phloem sap proteins qualitatively varying between WT and *bhlh121*, and +Fe and -Fe.

**Table S1:** list of primers used in this study.
